# Major Quantitative Trait Loci Control Low-Temperature Germination in Lettuce

**DOI:** 10.3390/life16030411

**Published:** 2026-03-03

**Authors:** Sunchung Park, Sookyung Oh, Ezekiel Ahn, Ainong Shi, Beiquan Mou

**Affiliations:** 1 Sustainable Perennial Crops Laboratory, Agricultural Research Service, United States Department of Agriculture, Beltsville, MD 20705, USA; ezekiel.ahn@usda.gov; 2 Environmental Microbial & Food Safety Laboratory, Agricultural Research Service, United States Department of Agriculture, Beltsville, MD 20705, USA; sookyung.oh@usda.gov; 3 Horticulture Department, University of Arkansas, Fayetteville, AR 72701, USA; ashi@uark.edu; 4 Sam Farr United States Crop Improvement and Protection Research Center, Agricultural Research Service, United States Department of Agriculture, Salinas, CA 93905, USA; beiquan.mou@usda.gov

**Keywords:** cold germination, quantitative trait loci, heritability, *Lactuca*, lettuce, seed

## Abstract

Low-temperature stress during germination is a major constraint for lettuce establishment in temperate and early-season production systems, causing delayed emergence, poor stand uniformity, and reduced yield. Cold germination represents an adaptive trait that enables seeds to initiate growth under suboptimal temperatures, but its genetic basis in lettuce remains poorly understood. Here, we investigated genetic architecture underlying cold germination using a biparental recombinant inbred line population derived from a cross between *Lactuca sativa* cv. Salinas and *Lactuca serriola* (wild lettuce). Phenotypic evaluations revealed substantial variation in germination performance at low temperatures, with cultivated lettuce exhibiting superior cold germination compared with the wild parent. Estimates of heritability indicated that genetic factors accounted for a large proportion of the observed phenotypic variation, demonstrating strong potential for selection. Quantitative trait locus (QTL) analysis identified two genomic regions significantly associated with cold germination ability, together explaining a substantial fraction of phenotypic variance (35%). These regions contained candidate genes involved in hormone signaling, membrane stability, and stress-responsive transcriptional regulation, including components of abscisic acid (ABA), gibberellic acid (GA), and ethylene pathways known to modulate germination under adverse conditions. Together, these results indicate that cold germination is a genetically complex trait that has likely been shaped through domestication and breeding. By elucidating the genetic basis of cold germination in lettuce, this study provides valuable targets for marker-assisted breeding aimed at improving seedling establishment and extending lettuce production into cooler environments.

## 1. Introduction

Successful seed germination is a critical developmental transition that determines seedling establishment, crop uniformity, and yield [1,2]. In temperate agricultural systems, low soil temperatures during early planting seasons pose a major challenge to germination, particularly for direct-seeded crops such as lettuce (*Lactuca sativa* L.). Although lettuce is classified as a cool-season crop with an optimal germination temperature around 18 °C [3], germination and emergence are markedly delayed at temperatures below 10 °C and are strongly suppressed at near-freezing conditions [4,5]. Slow or uneven emergence across the field under cold conditions reduces stand uniformity, prolongs canopy closure, and increases vulnerability to weed competition, ultimately limiting production efficiency and season extension [2,6]. In addition, uneven emergence leads to asynchronous maturity, which reduces yield in once-over harvesting systems used in most U.S. lettuce production, particularly for mechanically harvested baby leaf products [5].

Cold germination differs fundamentally from seed dormancy and thermoinhibition in both physiological basis and ecological significance. Seed dormancy prevents germination even under otherwise favorable conditions [7], whereas thermoinhibition suppresses germination at above-optimal temperatures [8,9,10]. In contrast, cold germination reflects a seed’s capacity to initiate metabolic and developmental processes under suboptimal thermal conditions, despite the kinetic and biochemical constraints imposed by low temperature [11,12]. This ability is particularly important for early spring planting and lettuce production in temperate regions where soil temperatures frequently fall below the optimal range for rapid emergence.

Previous studies have documented substantial phenotypic variation for cold germination among lettuce genotypes, including accessions from the USDA National Plant Germplasm System [5]. Some accessions are capable of germinating rapidly at temperatures as low as 5 °C, whereas others exhibit delayed or incomplete germination under the same conditions. However, the genetic mechanisms underlying this variation in lettuce remain largely unresolved. In contrast to thermoinhibition—where several key genes, including regulators of ABA biosynthesis and ethylene signaling, have been identified [8,13,14,15]—cold germination has received comparatively limited genetic dissection in lettuce.

Genetic variation for low-temperature germination has been more extensively studied in other vegetable species, including tomato, pepper, spinach, and cucumber, indicating that cold germination is a heritable trait amenable to genetic improvement [16,17,18,19]. Germination under suboptimal temperatures requires precise coordination of hormonal signaling pathways, among which the balance between abscisic acid (ABA) and gibberellins (GA) plays a central regulatory role [20]. ABA generally inhibits germination, whereas GA promotes endosperm weakening and radicle protrusion. In Arabidopsis thaliana, ABA signaling components such as ABI3 and ABI5 have been shown to suppress germination under stress conditions, including low temperature, while GA biosynthesis genes such as GA3ox and GA20ox promote germination by counteracting ABA-mediated repression [21,22]. Cold conditions often enhance ABA sensitivity and stabilize DELLA proteins, resulting in delayed germination [23]. Ethylene has also been demonstrated to promote germination under adverse conditions by antagonizing ABA action, and ethylene-insensitive mutants frequently exhibit reduced germination at low temperature [24,25]. In lettuce, ethylene responsiveness has long been recognized as a key factor influencing temperature-dependent germination, underscoring the importance of hormone crosstalk in regulating cold germination [8,13].

In addition to hormonal regulation, multiple physiological processes contribute to successful germination under low-temperature conditions. Cold stress imposes constraints on membrane integrity and cellular metabolism, making lipid biosynthesis and remodeling essential during early imbibition. Fatty acid desaturation and lipid metabolic pathways have been associated with improved membrane fluidity and enhanced germination under cold stress in multiple plant species [26,27]. Reactive oxygen species (ROS) also play a dual role during germination, functioning as signaling molecules required for radicle emergence while posing a risk of oxidative damage if not properly regulated. Controlled ROS accumulation and efficient antioxidant systems, including catalases and peroxidases, are therefore critical for germination under stress conditions [28]. Two-component signaling systems, including histidine kinases, further integrate hormonal and environmental cues and have been implicated in stress-responsive regulation during early plant development [29,30].

Domestication and breeding may have further shaped cold germination traits in lettuce. Modern cultivars have been selected for uniform emergence, yield, and performance under managed production environments, a process that can favor rapid and synchronized germination while potentially narrowing genetic diversity for stress-responsive germination traits. In contrast, wild relatives and landraces may retain alleles that enhance germination under unfavorable temperatures, reflecting adaptation to variable or unpredictable environments [2]. In tomato, for example, cultivated lines often exhibit improved low-temperature germination relative to primitive accessions [16], a pattern associated with reduced seed dormancy and altered hormonal sensitivity, particularly in ABA signaling [31]. Such domestication-driven shifts toward reduced dormancy and modified hormonal control may similarly underlie differences in germination behavior between cultivated lettuce and its wild progenitor, *Lactuca serriola*.

Together, these studies indicate that cold germination is a genetically complex trait governed by interactions between hormone signaling networks, metabolic pathways, and stress-response mechanisms. Despite growing insights from other crops, the genetic basis of cold germination in lettuce remains poorly characterized. Dissecting the genetic architecture underlying this trait through quantitative trait locus (QTL) analysis therefore provides a powerful approach to identify genomic regions and candidate genes contributing to cold germination. The pronounced difference in low-temperature germination between cultivated lettuce and wild lettuce, combined with the availability of genotyped recombinant inbred lines (RILs) derived from an interspecific *Lactuca sativa* × *Lactuca serriola* cross [32], make lettuce an attractive system for such analyses. By integrating phenotypic evaluation under low-temperature conditions with QTL analysis, this study aimed to (i) characterize variation in cold germination between domesticated and wild lettuce, and (ii) identify genomic regions and candidate genes associated with this trait, thereby providing a genetic foundation for improving early-season establishment and extending lettuce production into cooler environments.

## 2. Materials and Methods

### 2.1. Plant Materials and Cold Germination Phenotyping

An interspecific RIL population consisting of 153 lines, derived from a cross between the cultivated lettuce (*Lactuca sativa*) cv. Salinas and wild lettuce accession US96UC23 (*Lactuca serriola*) [32] was used for cold germination phenotyping. Cold germination assays were performed under controlled-environment conditions using a growth chamber. For each RIL, 25 seeds without sterilization treatment were sown onto distilled water-moistened Whatman Grade 1 filter paper placed in 100 × 15 mm Petri dishes (Fisher Scientific, Waltham, MA, USA). Petri dishes were arranged in a randomized complete block design within the germination chamber and maintained under a 12 h light/12 h dark photoperiod. Cold germination was evaluated at a constant 5 °C, while control germination was conducted at 21 °C. Light intensity was maintained at 100 µmol m^−2^ s^−1^ for both conditions. To maintain high humidity, open Petri dishes containing distilled water were placed on the chamber shelves. Germination was assessed 18 days after imbibition, with seeds exhibiting radicle protrusion of at least 2 mm and fully opened cotyledons scored as successfully germinated (Figure S1). To minimize positional effects within the chamber, petri dishes were rotated daily throughout the experiment. The assay was conducted with three replicates, consisting of three independent experimental runs performed on different dates. Cold germination performance was quantified as the ratio of germination at 5 °C to germination at 21 °C for each line.

### 2.2. Quantitative Trait Locus Analysis

QTL mapping for cold germination was conducted using 153 RILs that had previously been genotyped with the lettuce 6.6 million feature Affymetrix high density GeneChip at the University of California, Davis, CA, USA (https://michelmorelab.ucdavis.edu). The resulting genetic map and phenotype used for QTL analysis is provided in Supplementary Table S1 and Table S2, respectively. Physical positions of the single-position polymorphic markers were updated to the lettuce reference genome assembly version 8 (NCBI accession GCF_002870075.4) by aligning marker nucleotide sequences to the genome using BLASTN (Table S3).

Because low-temperature germination (LTG) represents proportion data, phenotypic values were transformed prior to analysis to better satisfy normality assumption. A probit transformation was applied using the *probitlink* function of the VGAM R package v1.1 [33]. Normality of the phenotypic distribution was evaluated using the Shapiro–Wilk test [34]. The transformation substantially improved normality (W increased from 0.923, *p* = 2.9 × 10^−7^ to 0.963, *p* = 4 × 10^−3^). Although minor deviations from normality remained, the transformed data provided a closer approximation to a normal distribution than the untransformed values and were therefore used for all downstream QTL analysis.

QTL mapping was performed with the R/qtl package v1.70 [35]. Significance thresholds for logarithm of the odds (LOD) scores were estimated by 1000 permutation testing of the phenotypic data, with all markers included and no additional additive or interactive covariates specified. Model selection penalties were calculated from the permutation results using the *calc.penalties* function at multiple significance levels (α = 0.10, 0.05, 0.01, and 0.001). A stringent penalty threshold of α = 0.01 was selected for subsequent analyses to control model complexity and minimize the detection of spurious QTL.

QTLs were identified using a stepwise model selection approach implemented in R/qtl (*stepwiseqtl*), corresponding to multiple interval mapping (MIM). Haley–Knott regression was used for likelihood estimation, and the maximum number of QTL allowed in the model was set to eight. Both additive and pairwise epistatic interactions were evaluated during model fitting [36]. The final QTL model was evaluated using the *fitqtl* function to estimate LOD scores and the percentage variation explained (PVE) by each QTL.

Candidate genes underlying each QTL were identified based on physical positions within the Bayesian 95% credible interval for each QTL, using gene annotations from the lettuce reference genome v8.

### 2.3. Functional and Gene Ontology (GO) Annotation of Candidate Genes

Functional annotation of the candidate protein set was performed using the Trinotate pipeline [37], as described by Park et al. [38]. Briefly, predicted protein sequences of candidate genes were queried against the UniProtKB/Swiss-Prot database using BLASTP with an E-value cutoff of 1 × 10^−5^. In parallel, protein sequences were analyzed against the Pfam database using the *hmmsearch* tool of HMMER [39] to identify conserved domains. Functional annotations and GO terms were assigned to candidate proteins based on sequence similarity from UniProtKB/Swiss-Prot entries (Table S4).

To assess whether sequence divergence may underlie functional differentiation of candidate genes between cultivated and wild lettuce, candidate protein sequences from *L. sativa* were compared with predicted protein sequences from the genome assembly of wild lettuce (*L. serriola* accession US96UC23; NCBI accession GCA_051521515.1) using BLASTP with an E-value threshold of 1 × 10^−50^. This comparative analysis enabled the identification of amino acid-level differences, including substitutions, insertions, deletions, and potential gene loss between the two species.

### 2.4. Heritability and QTL Variance Estimation

To estimate genetic contributions to variation in cold germination, both broad-sense and narrow-sense heritability were calculated using linear mixed models. Broad-sense heritability (H^2^) was estimated using a mixed-effect model that partitions phenotypic variance into among-line and residual components: y=Xβ+g+e (*y*: the phenotypic observation, Xβ: fixed effects (replication), g: the random effect of line, and e: the residual error). The random line effect was assumed to follow $g∼N(0,\sigma_{G}^{2})$, and residuals were assumed independent with variance $\sigma_{E}^{2}$. Broad-sense heritability was calculated as ${H^{2}=\sigma }_{G}^{2}/(\sigma_{G}^{2}+\sigma_{E}^{2}/r)$ ($\sigma_{G}^{2}:$ genetic variance among lines, $\sigma_{E}^{2}$: residual variance, and r: number of replications per line).

Narrow-sense heritability was estimated using a genomic linear mixed model incorporating genome-wide marker information to capture additive genetic effects. A genomic relationship matrix (G) was constructed from genetic markers to represent additive genetic relatedness among RILs. The model was specified as $y=X\beta +g+e,\;g∼N\left(0,G\sigma_{A}^{2}\right),\;e∼N(0,I\sigma_{E}^{2})$ (g: vector of additive genetic effects, G: genomic relationship matrix, $\sigma_{A}^{2}:$ additive genetic variance, I: identity matrix, and $\sigma_{E}^{2}:$ residual variance). Narrow-sense heritability was calculated as $h^{2}=\sigma_{A}^{2}/{(\sigma }_{A}^{2}+\sigma_{E}^{2}/r)$. Variance components were estimated using restricted maximum likelihood (REML). All mixed models were fitted using the *sommer* package in R [40].

To quantify the contribution of the two detected QTLs (markers BAVS and BUYM) to phenotypic variation, a multiple-QTL linear model was fitted using line-mean phenotypic values. QTL genotypes were coded additively (A = −1, B = +1), and missing genotypes were imputed using the marker mean. The full model was specified as $y=X\beta +Q_{1}+Q_{2}+e$, where $Q_{1}$ and $Q_{2}$ represent the additive effects of the two QTLs. A reduced (baseline) model including only replication effects was also fitted. The phenotypic component attributable exclusively to the two QTLs was obtained by subtracting fitted values of the baseline model from those of the full model. The variance explained by the two QTLs was estimated as the variance of this QTL-attributable component. The proportion of phenotypic variance explained by the two QTLs on a line-mean basis was calculated as: ${\mathrm{PVE}}_{2QTL}=\sigma_{QTL}^{2}/\left(\sigma_{QTL}^{2}+\sigma_{E}^{2}/r\right)$, where $\sigma_{QTL}^{2}$ is the variance explained by the two QTLs and $\sigma_{E}^{2}$ is the residual variance estimated from the full model. This metric represents the fraction of variation among line means attributable to the two detected loci and was compared with genome-wide narrow-sense heritability to assess the proportion of additive genetic variance captured by the major QTLs.

## 3. Results

### 3.1. Genotype Effects on Cold Germination

**Figure 1:**
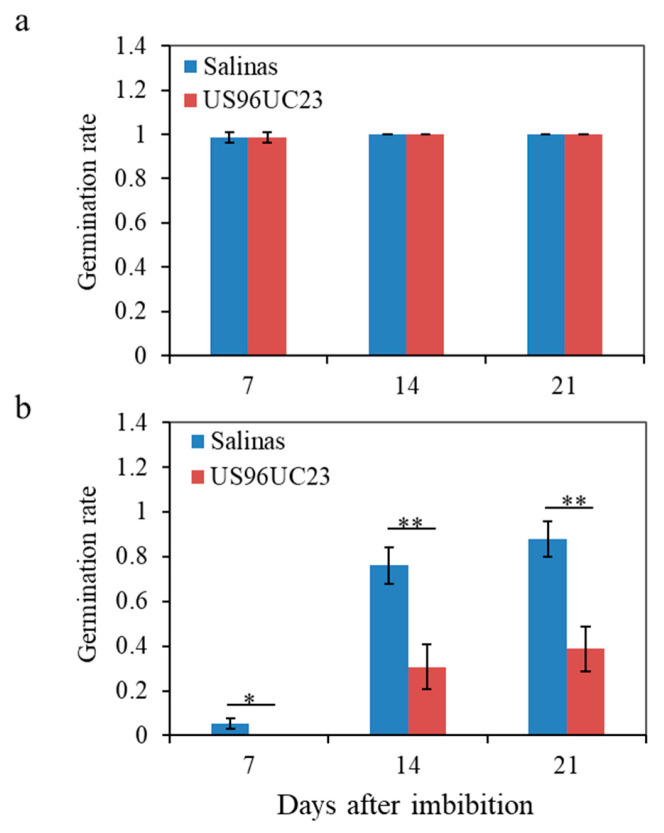
Germination rates of *L. sativa* cv. Salinas and *L. serriola* cv. US96UC23 under warm (21 °C) and cold (5 °C) conditions. Germination rate (*y*-axis) was calculated as the proportion of seeds that successfully germinated relative to the total number of seeds tested under warm (**a**) and cold (**b**) conditions. Germination was assessed at 7, 14, and 21 days after imbibition. Error bars indicate mean ± standard deviation. Asterisks denote statistical significance between genotypes at each time point determined by two-tailed *t*-tests (*p* < 0.05 (*); *p* < 0.01 (**)).

Germination under cold and warm conditions was evaluated for the cultivated lettuce cv. Salinas and the wild lettuce accession US96UC23. To assess the effects of genotype and time on germination, a two-way analysis of variance (ANOVA) was performed with genotype (Salinas and US96UC23) and time (7, 14, and 21 days after seed imbibition) as fixed factors (Figure 1). Under cold conditions, the analysis revealed a highly significant effect of genotype (*p* < 0.0001), indicating that Salinas consistently exhibited higher germination than US96UC23 across all time points (Figure 1b). In contrast, no significant genotype effect was detected under warm conditions, suggesting that the difference between the two genotypes was specific to low-temperature germination (Figure 1a). A strong effect of time was also observed for cold germination (*p* < 0.0001), reflecting a progressive increase in germination from 7 to 14 and 21 days after imbibition, regardless of genotype. Importantly, the genotype × time interaction was significant (*p* = 0.0006), indicating that the temporal dynamics of cold germination differed between the two genotypes, with Salinas showing a more pronounced increase in germination over time compared with US96UC23.

Given the significant genotype × time interaction, pairwise comparisons between genotypes were conducted separately at each time point under cold conditions using two-sample *t*-tests. At 7 days, Salinas already exhibited significantly higher germination than US96UC23 (*p* = 0.016), indicating an early divergence between the two genotypes (Figure 1b). This difference became more pronounced at 14 days (*p* = 0.0036). A similar strong difference was maintained at 21 days (*p* = 0.0027), confirming that the germination advantage of Salinas persisted throughout the evaluation period. Together, these results demonstrate that Salinas not only maintains higher values than US96UC23 but also exhibits a faster and more robust temporal increase in germination under low-temperature conditions.

### 3.2. Identification of QTL Controlling Cold Germination

**Figure 2:**
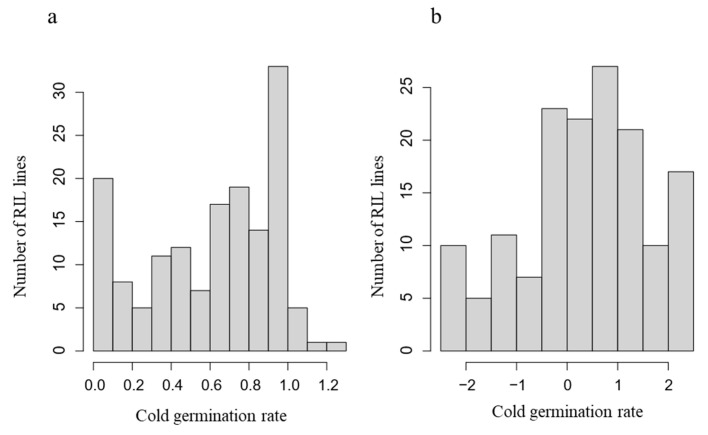
Distribution of cold germination rate in the RIL population based on raw data (**a**) and probit-transformed data (**b**). Cold germination rate was calculated as the ratio of seeds germinated at 5 °C relative to 21 °C at 18 days after imbibition.

Because ANOVA revealed a significant genotype effect on cold germination, QTL analysis was conducted using the RIL population derived from a cross between cv. Salinas and US96UC23 (Figure 2). The distribution of low-temperature germination (LTG) values deviated from normality (Figure 2a). Therefore, the data were transformed using a probit transformation, which substantially improved the approximation to a normal distribution (Figure 2b). The transformed values were used for all subsequent QTL analyses.

QTL mapping identified genomic regions significantly associated with variation in cold germination. The final multiple-QTL model was highly significant, explaining 34.9% of the total phenotypic variance (PVE) and showed strong statistical support based on both chi-square and *F*-tests (χ^2^ *p* = 5.2 × 10^−15^; *F*-test *p* = 1.0 × 10^−15^) (Table 1). The model accounted for a sum of squares of 85.1 with 2 degrees of freedom, whereas the residual error accounted for a sum of squares of 158.4 across 150 degrees of freedom, resulting in a high overall LOD score of 14.3. These results indicate the presence of major genetic factors contributing to variation in cold germination.

**Figure 3:**
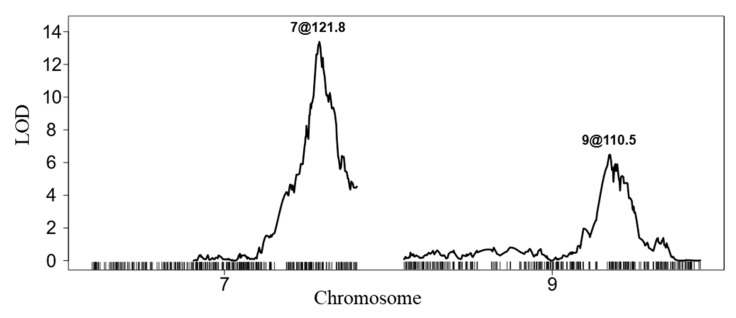
QTL analysis identifying genomic regions associated with cold germination. Two major QTL peaks were detected at 121.8 cM on chromosome 7 and 110.5 cM on chromosome 9, indicating loci contributing to variation in germination under low-temperature conditions. The *y*-axis represents the logarithm of the odds (LOD) score. Genome-wide significance threshold for main effects (LOD = 4.20) and interaction effects (LOD = 5.58) were established based on 1000 permutation tests.

Consistent with the strong support for the multiple-QTL model, two significant QTLs were detected (Figure 3; Table 2). The primary locus, QLTG7.1, was mapped to chromosome 7 at 121.8 cM (marker BAVS) and exhibited a LOD score of 13. The position of qLTG7.1 was well supported by both a 95% Bayesian credible interval (120.2–123.6 cM) and a 1.5-LOD support interval (119.7–123.9 cM), defining a relatively narrow confidence region. qLTG7.1 displayed a large additive effect (0.45) and explained 25.8% of the phenotypic variance, identifying it as a major-effect locus underlying cold germination.

A second QTL, qLTG9.1, was detected on chromosome 9 at 110.5 cM (BUYM), with a LOD score of 6 (Table 2). The confidence interval for qLTG9.1 was broader than that of qLTG7.1, spanning 103.6–117.5 cM based on the 95% Bayesian credible interval and 103.6–117.8 cM using the 1.5-LOD support interval. qLTG9.1 exhibited a moderate additive effect (0.33) and accounted for 13.5% of the phenotypic variance, indicating a secondary but significant contribution to cold germination.

Together, qLTG7.1 and qLTG9.1 explained a substantial proportion of the observed phenotypic variation, suggesting that cold germination in lettuce is governed by a combination of a major-effect locus and an additional moderate-effect locus.

### 3.3. Candidate Genes Underlying Cold Germination QTL

**Figure 4:**
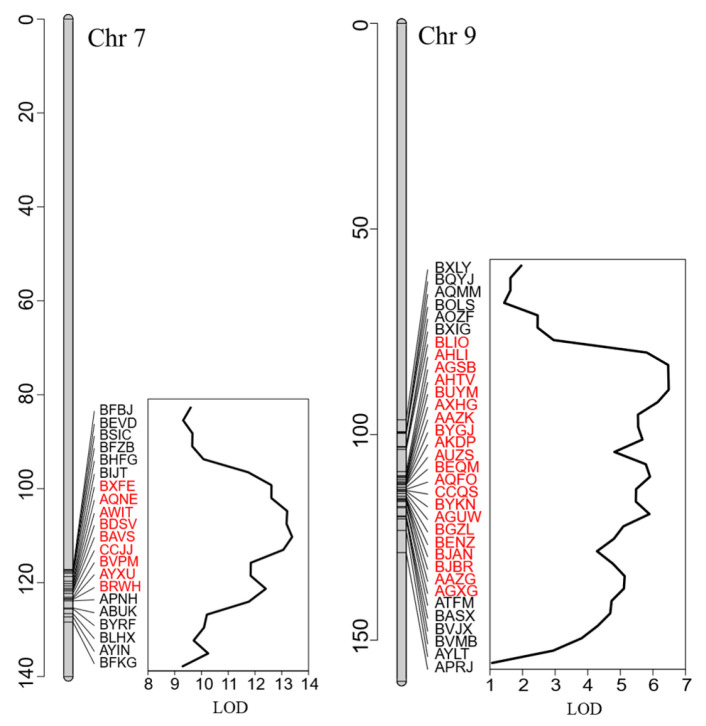
Chromosomal locations of the two QTLs associated with cold germination in lettuce. The positions of qLTG7.1 on chromosome 7 and qLTG9.1 on chromosome 9 are shown on their respective genetic linkage maps, with positions in centimorgans (cM; *y*-axis) and markers names indicated. Line plots represent LOD scores across markers along each chromosome. Gray bars depict chromosomes, and tick marks denote marker positions on the linkage maps. Red-highlighted markers define the 95% Bayesian credible intervals for each QTL.

Candidate genes were identified within the 95% Bayesian credible intervals of the detected cold germination QTLs. qLTG7.1 was flanked by markers BXFE and BRWH, which are physically located at 163,756,084 and 167,324,102 on chromosome 7, respectively (Figure 4; Table S3). This interval spans approximately 3.57 Mb and contains 106 protein-coding genes. The second QTL, qLTG9.1, was flanked by markers BLIO and AGXG, located at 145,742,377 and 159,040,992 bp on chromosome 9, respectively (Figure 4; Table S3). The qLTG9.1 interval spans approximately 13.3 Mb and contains 243 protein-coding genes.

Functional annotation revealed that many genes within both QTL intervals were associated with hormone signaling, transcriptional regulation, and stress responses. Notably, genes related to ABA signaling, GA metabolism, ethylene biosynthesis, auxin, and abiotic stress response were identified, consistent with established regulatory mechanisms controlling seed germination under adverse environmental conditions (Table S4). In addition, a subset of genes associated with seed development was identified, resulting in a total of 29 candidate genes prioritized for further analysis (Table S5).

To assess potential functional divergence between cultivated and wild lettuce, protein sequences of candidate genes from *L. sativa* were compared with predicted protein sequences from the *L. serriola* genome. This analysis aimed to identify candidate genes that may differ between two species due to sequence divergence or gene loss and that could therefore contribute to their contrasting cold germination phenotypes. Approximately half of the candidate proteins from *L. sativa* showed identical matches in *L. serriola*, whereas 13 candidate proteins showed sequence divergence, with their closest *L. serriola* homologs differing by 1 to 16 amino acids and/or displaying N-terminal or C-terminal truncations (Table S5).

Notably, three candidate proteins did not yield significant matches in the *L. serriola* proteome using a stringent BLASTP E-value cutoff of 1 × 10^−50^, suggesting potential gene loss or substantial divergence following species separation (Table S5). These genes were annotated as Anaphase-promoting complex subunit 1, bifunctional 3-dehydroquinate dehydratase/shikimate dehydrogenase (chloroplastic), and SUPPRESSOR OF PHYA-105 1. These genes are associated with abiotic stress responses and seed developmental processes and are therefore plausible contributors to cold germination differences between cultivated and wild lettuce. Their potential functional roles in regulating cold germination are discussed further in Section 4.

### 3.4. Heritability

Both broad-sense and narrow-sense heritability estimates indicated strong genetic control of cold germination in the RIL population. Broad-sense heritability was high (H^2^ = 0.901), indicating that the majority of phenotypic variation among RILs was largely attributable to genetic differences rather than environmental or residual effects. Genome-wide narrow-sense heritability was also substantial (h^2^ = 0.711), suggesting that additive genetic effects accounted for a large proportion of the total genetic variance underlying cold germination.

The two major QTLs identified for this trait explained a considerable fraction of the observed variation, although the estimated contribution differed depending on the analytical framework. Based on ANOVA of individual observations (Table 1), which partitions total phenotypic variance including residual and environmental components, the two loci together accounted for approximately 35% of the total phenotypic variance. In contrast, when evaluated using a linear model on a line-mean basis–thereby reducing environmental noise through replication and focusing on genetic differences among lines—the two QTLs jointly explained 57% of the variance among line means. The higher variance explained on a line-mean basis reflects the removal of within-line environmental variation and provides a measure that is directly comparable to narrow-sense heritability. The close correspondence between narrow-sense heritability and the additive variance explained by the detected QTL indicates that a large proportion of the additive genetic variance for cold germination is captured by these two loci.

## 4. Discussion

### 4.1. Evolutionary and Ecological Divergence of Temperature Responses

Low-temperature stress during germination remains a major barrier to reliable lettuce establishment in temperate and early-season production systems. Our study demonstrates that cold germination exhibits substantial genetic regulation and is under polygenic genetic control, with enhanced performance observed in cultivated *L. sativa* compared to wild *L. serriola* accessions. This pattern contrasts with the thermoinhibition response observed under high-temperature conditions, where *L. serriola* exhibited more robust germination than cultivated lettuce [10,31]. These opposing responses across temperature regimes suggest that germination behavior in lettuce might have been shaped by both domestication and subsequent artificial selection, rather than reflecting a uniform stress-tolerance strategy [41].

Enhanced germination of wild *L. serriola* under high temperatures is consistent with its ecological origin and life-history as the wild species adapted to disturbed and often unpredictable environments [42,43]. Germination at elevated temperatures may facilitate rapid establishment during warm periods, while suppressed germination at low temperatures likely serves as a risk-avoidance mechanism that prevents emergence under early-season cold conditions that could compromise seedling survival, a pattern consistent with adaptive dormancy that prevents emergence during unfavorable cold conditions. Temperature-sensitive dormancy responses of this type are well documented in wild plant species [2,31] and are frequently mediated by heightened sensitivity to abscisic acid and environmental cues [44].

In contrast, cultivated lettuce displayed improved germination under low-temperature conditions but reduced performance under heat stress. This pattern is consistent with the effects of domestication and breeding for agricultural production systems, where rapid and uniform emergence under cool conditions is highly desirable [2,45]. Selection for early planting, synchronized emergence, and reliable stand establishment in temperate climates has likely favored alleles that reduce dormancy and broaden the lower thermal threshold for germination. At the same time, relaxation of selection for heat-adaptive germination responses may explain the comparatively weaker performance of cultivated lettuce under high temperatures. This trade-off suggests that breeding for cold germination may have inadvertently narrowed the upper thermal window for optimal germination.

Together, these findings highlight how domestication and breeding have reshaped germination temperature responses in lettuce. Superior cold germination in cultivated lettuce likely reflects selection during domestication and subsequent breeding that contributes to modern agricultural performance, whereas enhanced heat germination of wild lettuce reflects ecological fitness rather than agronomic value. Understanding this divergence provides valuable insight into the evolutionary and genetic basis of germination traits and offers opportunities to strategically combine favorable alleles from wild and cultivated germplasm to improve germination stability across a broader range of temperature conditions.

### 4.2. Genetic Architecture and Heritability of Cold Germination

The QTL identified in this study appear to be genetically distinct from previously characterized thermoinhibition loci such as *LsNCED4* [31], which has been shown to regulate high-temperature germination inhibition in lettuce. The chromosomal positions of qLTG7.1 and qLTG9.1 do not overlap with the reported location (62–78 cM on Chr 6) of *LsNCED4*, suggesting that cold germination and thermoinhibition are controlled by independent genetic mechanisms. This distinction highlights the novelty of the loci identified here and underscores the complexity of temperature-dependent germination regulation in lettuce.

The high broad-sense heritability (H^2^ = 0.9) observed for low-temperature germination indicates that phenotypic variation in this RIL population is predominantly determined by genetic factors, with environmental and measurement effects contributing relatively little to overall variation. The high repeatability across replications underscores the robustness of the phenotyping and supports the reliability of subsequent genetic analyses. The substantial narrow-sense heritability (h^2^ = 0.71) further indicates that additive genetic effects represent the dominant component of the genetic variance for this trait, suggesting that selection based on additive effects should be effective and that low-temperature germination is highly amenable to genetic improvement.

Although additive effects predominate, the difference between broad- and narrow-sense heritability implies that non-additive genetic effects, such as epistasis, also contribute to phenotypic variation, albeit to a lesser extent. Such a pattern is characteristic of many stress-related traits, in which additive loci provide the primary response to selection while epistatic interactions modulate trait expression and fine-tune phenotypic outcomes [16,31,46,47].

Consistent with this genetic architecture, the two QTLs identified in this study together explained a substantial proportion of the phenotypic variance for cold germination. The linear model–based estimate of variance explained (57%), which incorporates the joint effects of both loci and explicitly accounts for replication, is directly comparable to the genome-wide narrow-sense heritability. Comparison of QTL-based PVE with the narrow-sense heritability indicates that these two loci account for approximately 80% (0.57/0.711) of the additive genetic variance in the population. Collectively, these results suggest that low-temperature germination in this RIL population is largely controlled by a small number of moderate- to large-effect loci, with additional minor-effect loci and non-additive interactions contributing to the remaining genetic variation. This genetic architecture—characterized by major QTL embedded within a polygenic background—provides clear and actionable targets for marker-assisted selection, while retaining potential for further genetic gains through background improvement and recombination.

### 4.3. Candidate Genes and Breeding Implications

The identification of hormone- and stress-related candidate genes within cold germination QTL highlights the central role of ABA, GA, and ethylene signaling pathways in mediating germination responses under low temperature conditions. These pathways are well known to coordinate metabolic activation, embryo growth, and endosperm weakening during germination, particularly under environmental stress [21,22,44]. The enrichment of genes associated with these signaling networks provides a genetic framework for improving lettuce cold germination through marker-assisted selection and targeted introgression from wild or exotic germplasm. Notably, three candidate genes lacked detectable homologs in *L. serriola*, suggesting lineage-specific divergence or acquisition during domestication that may have contributed to the superior cold germination observed in cultivated lettuce.

One such candidate is Anaphase-promoting complex subunit 1 (APC1), a core component of the anaphase-promoting complex/cyclosome (APC/C), a conserved E3 ubiquitin ligase that regulates cell-cycle progression through targeted proteasomal degradation of key regulatory proteins [48]. Successful germination—especially under low-temperature conditions—requires tight control of cell cycle re-entry and coordination between embryo growth and endosperm weakening [49]. Because cold stress slows metabolic and mitotic processes, premature entry into the cell cycle can be detrimental, making tight regulation of cell-cycle checkpoints especially critical during early germination. Variation in APC/C activity may therefore influence cold germination by modulating the timing of embryo cell division under low-temperature conditions.

SUPPRESSOR OF PHYA-105 1 (SPA1) is a key regulator of phytochrome A (PhyA) signaling, and plays an important role in integrating light, temperature, and hormonal cues during seed germination [50]. SPA1 modulates CONSTITUTIVELY PHOTOMORPHOGENIC 1 (COP1) activity, thereby influencing photomorphogenesis and hormone sensitivity [50]. Given the extensive crosstalk between phyA signaling, ABA and GA pathways, and temperature perception during germination [2,22,51], SPA1 represents a compelling candidate gene underlying cold germination. Altered SPA1-mediated regulation could affect the integration of environmental and hormonal signals required for germination under low-temperature conditions.

A third candidate encodes bifunctional 3-dehydroquinate dehydratase/shikimate dehydrogenase, a key enzyme of the shikimate pathway responsible for the biosynthesis of aromatic amino acids, including phenylalanine, tyrosine, and tryptophan [52]. These amino acids serve as precursors for auxin, phenylpropanoids, and a wide range of antioxidant compounds. Cold stress is frequently associated with increased production of ROS, necessitating efficient antioxidant defenses to maintain cellular integrity during germination [53]. Shikimate-derived metabolites contribute to redox homeostasis, membrane stabilization, and stress signaling [52], suggesting that variation in this pathway may enhance stress resilience during early seedling establishment. Together, these candidate genes represent potential molecular targets for breeding strategies aimed at improving lettuce establishment under cool and variable temperature conditions.

We hypothesize that allelic variation in SPA1 may alter phytochrome A signaling and its crosstalk with ABA and GA pathways during cold imbibition. This hypothesis can be tested by analyzing differential gene expression between parental lines during early stages of cold germination. Similarly, variation in APC1 may influence the timing of cell-cycle reactivation under low-temperature conditions. This could be evaluated through transcriptomic profiling and cell-cycle marker analysis during early imbibition at 5 °C. We further hypothesize that allelic variation in the shikimate pathway enzyme may influence cold germination by modulating metabolic flux, thereby affecting downstream production of auxin, phenylpropanoids, and antioxidant compounds. This can be tested by comparing gene expression levels, metabolite profiles (e.g., phenylalanine and flavonoids), and ROS accumulation between parental lines during early germination at 5 °C.

The flanking markers BAVS (qLTG7.1) and BUYM (qLTG9.1) provide practical tools for marker-assisted selection (MAS). These markers can facilitate the introgression of superior low-temperature germination alleles into elite lettuce germplasm, thereby accelerating breeding efforts aimed at improving early-season establishment and extending production into cooler environments.

Most U.S. lettuce crops are produced in California, Arizona, and Florida, where mild winter climates enable year-round production. In contrast, lettuce production in other states is constrained by shorter growing seasons. However, demand for local leafy vegetable production in these regions is increasing due to rising transportation costs from coastal production areas and growing interest in reducing carbon footprints. Collectively, these findings provide a genetic framework for enhancing lettuce germination under cold soil conditions, with potential to extend production into cooler regions and contribute to more regionally distributed vegetable systems.

## Figures and Tables

**Table 1:** ANOVA results for the final additive QTL model for cold germination.

Source	df	Sum of Squares	Mean Square	LOD	PVE (%)	*p*-Value (Chi2)	*p*-Value (*F*)
Model	2	85.1	42.5	14.3	34.9	5.2 × 10^−15^	1 × 10^−15^
Error	150	158.4	1.1				
Total	152	243.4					

Note: df, degrees of freedom; LOD, logarithm of the odds; PVE, percent variance explained. *p*-values were obtained from Chi-square and *F* tests based on the fitted QTL model in a RIL population.

**Table 2:** QTL for variation in cold germination.

QTL	Chr	Pos (cM)	LOD	Bayesian 95% CI	1.5 LOD CI	Effect Size	PVE
qLTG7.1	7	121.8	13	120.2–123.6	119.7–123.9	0.45	25.8
qLTG9.1	9	110.5	6	103.6–117.5	103.6–117.8	0.33	13.5

Entries include map positions (Pos) in centiMorgan (cM), LOD score, 95% Bayesian credible interval (CI), additive effect sizes, and percent variance explained by each QTL (PVE). Effect sizes represent the difference in mean germination between genotypes homozygous for the *L. sativa* cv. Salinas and *L. serriola* (US96UC23) allele, respectively; Positive values indicate that the *L. sativa* allele is associated with higher cold germination.

## Data Availability

The original contributions presented in this study are included in the article/Supplementary Material. Further inquiries can be directed to the corresponding author.

## Supplementary Materials

The following supporting information can be downloaded at https://www.mdpi.com/article/10.3390/life16030411/s1. Table S1: Genotypes of the RIL population derived from a cross between *L. sativa* cv. Salinas and *L. serriola* accession US96UC23; Table S2: Phenotype of the RIL population derived from a cross between *L. sativa* cv. Salinas and *L. serriola* accession US96UC23; Table S3: Physical locations of genetic markers used in QTL analysis; Table S4: Functional and Gene Ontology (GO) annotation of Candidate Genes underlying the cold-germination QTL in lettuce; Table S5: Selected Candidate Genes Involved in Hormone Signaling, Seed Development, and Abiotic Stress within Cold-Germination QTLs; Figure S1: Representative images illustrating germination scoring criteria.

## Abbreviations

The following abbreviations are used in this manuscript:

ABA	Abscisic acid
APC1	Anaphase-promoting complex subunit 1
APC/C	Anaphase-promoting complex/cyclosome
GA	Gibberellic acid
GO	Gene Ontology
H^2^	Broad-sense heritability
h^2^	Narrow-sense heritability
LOD	Logarithm of the odds
LTG	Low-temperature germination
PVE	Phenotypic variance explained
QTL	Quantitative trait locus
REML	Restricted maximum likelihood
RIL	Recombinant inbred line
ROS	Reactive oxygen species
SPA1	SUPPRESSOR OF PHYA-105 1
