# Supplementary material for: Major Quantitative Trait Loci Control Low-Temperature Germination in Lettuce
Source: Life (Basel). 2026 Mar 3;16(3):411. doi: 10.3390/life16030411 (PMC13028493; doi:10.3390/life16030411)
Supplement: Supplementary file 1 [file life-16-00411-s001.zip › Figure S1.pdf]

Article

Major Quantitative Trait Loci Control Low-Temperature Germination in Lettuce

Sunchung Park [1,*], Sookyung Oh [2], Ezekiel Ahn [1], Ainong Shi [3], and Beiquan Mou [4]

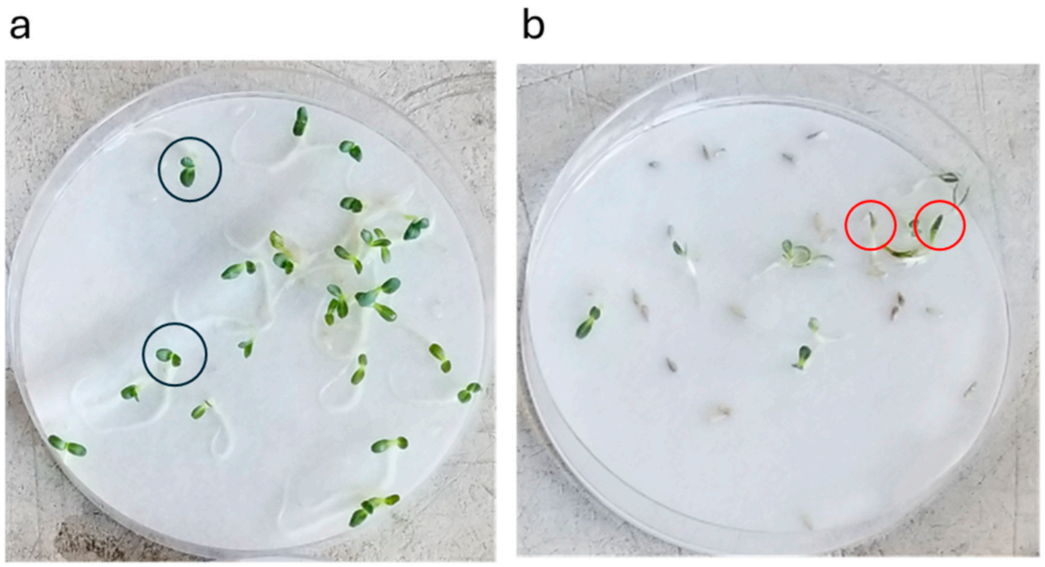

Figure S1. Representative images illustrating germination scoring criteria. Twenty-five seeds were germinated on Petri dishes containing wetted Whatman filter paper. Germination was considered successful when cotyledons were opened marked (black circle (a)). Seeds that had germinated but exhibited unopened cotyledons were scored as unsuccessful (red circles (b)).
